# Everybody's Page

**Published:** 1889-05-04

**Authors:** 


### Everybody's Page.

There are 2,500 medical women in the United States.

M. Verneuil recently expressed the opinion before the Académie de Médecine of Paris that tetanus is an infectious disease of equine origin.

The latest treatment recommended for diphtheria comes from Königsberg, where Dr. Arthur Hening has had very successful results from the use of limewater and ice bags.

St. John's, Newfoundland, has just established a system of compulsory insurance for fishermen. Each boat-owner pays fifty cents for each man in his employ at the beginning of every voyage, and the men themselves pay twenty cents. If any of these fishermen lose their lives at sea, the widows are entitled to a small annuity.

"Manners," says the *Journal d'Hygiene*, "are the foundation of hygiene." The first step in sanitation is personal cleanliness, and that is purely a matter of custom. The daily ablutions of a Spaniard would hardly satisfy an Englishman, and there are still boarding-schools in France where if a pupil asked for a bath, the teacher would exclaim, "A bath! Are you ill?"

From Mr. Arthur Smith's article in the *National Review*, it seems that plants have what may truly be defined as brain-power—and perhaps consciousness as well. If that be so, Mr. Charles Collette's anecdote of his father's cauliflower—"a tame cauliflower that was fond of him and followed him about like a dog"—may be regarded as something more than a laughable absurdity, and the "language of flowers" may one day, in the progress of evolution, become audible.

A travelling show recently exhibited a snake's skin, to which the following interesting legend was attached: "Skin of the serpent that tempted Eve in the Garden of Eden. It was killed by Adam the day after the Fall. Adam hit it with a club, the traces of which are still left. This skin was part of the inheritance of Adam, and was preserved in his family in Asia. The genuineness is attested by doctors of divinity, whose seals are attached." This is, indeed, a treasure for the antiquary!

It is well known that Boston is the most "cultured" town in America, if not in the world, and a true Bostonian would rather take a box on the ear than have that organ offended by a mispronunciation. A native of that favoured spot having had occasion to call in a doctor, his wife asked him, "What did the doctor pronounce your ailment?" "He pronounced it as if it was spelled 'bronkeetus,'" exclaimed the indignant sufferer; "so I at once requested him to tell me his fee and go."

It is rather strange that tobacco, which is, after all, the most harmlesss of stimulants, should be so violently hated by some good folks. Recently a prominent religious sect in America promulgated a manifesto to the following effect: "Whereas the use of tobacco in any shape or form is a filthy, pernicious, and degrading habit, imperilling the health of the body and the welfare of the soul alike, leading to a laxity in living which is the sure road to intemperance and all vice"—and more to the same effect—"Therefore, it is resolved that this conference will not grant ordination or license to preach to any candidate who is a victim to this debasing habit." How are we to regard this awful fulmination? As smoke, most grave and reverend seignors—nothing but smoke.

An American newspaper thus records the death of a medical man: "We have to lament the death of Dr. S——, of C——. He died a martyr to duty; forgetful of self and his own sufferings. A loyal, Christian man and physician. His practice is for sale. Address his widow, Mrs. E. J. S——."

Moorhnol, a very bitter aromatic oil obtained by extracting cod liver oil with 90 per cent. alcohol and distilling the product, is now being strongly recommended. It contains bromine, iodine, and phosphorus, and it is said that a morrhnol capsule of three grains is equal in therapeutical value to about 1½ drachms of cod liver oil.

A patent has been taken out in France for preserving seed and plants from the action of parasites and hastening the germination of the seeds. To protect them when placed in the ground they are steeped in antiseptic and fertilising fluids. The best liquid is said to be composed as follows: Acetate of aluminium, 1[illegible] parts; acetate of lead, 70 parts; carbonate of lime, 15 parts. These are dissolved in water, and used either for the seed, or for watering the young plants.

The manufacture of artificial teeth approaches to the proportion of a great industry in America. Three houses alone produced 20,000,000 teeth last year. Artificial teeth cannot, as might be thought, be turned out all alike; delicate variations of colour have to be made to suit the tastes of various countries. For example: Canadians need their teeth white, while in South America the demand is for teeth of a yellowish tint, and in China nothing will sell but black teeth.

The epicure sees with regret the advent of the months with no "R" in them, for in May, June, July, and August oysters are out of season. So does the doctor, for there is no dainty likely to tempt a patient's appetite that has the nutritive value of the oyster. A quart of oysters contains as much nutritive substance as a quart of milk, or a pound of lean beef, or a pound and a half of fish, or two-thirds of a pound of bread. Oysters are rich both in the flesh-forming compounds which make muscle, blood, bone, and brain, and in the ingredients which are changed in the body into heat and force. Oysters are more nearly equivalent to milk than almost any food, and they are often enjoyed and assimilated when milk would be rejected.

A good many years ago a remedy for sleeplessness, invented by one Mr. Gardner, attracted a good deal of attention, and many people testified to its efficacy. The inventor himself suffered from insomnia, the result of a severe injury to the spine, caused by being thrown out of a carriage. His prescription was as follows: First, lie on the right side, with the head placed comfortably on the pillow, and the neck straight, so that respiration may go on easily. A full inspiration being taken, the sufferer is to give no further heed to the action of his lungs, but is to devote all his energies to imagining that he sees his breath issuing from his nostrils in a continuous stream, as one does from a horse's on a frosty day. As soon as he could bring himself to imagine this apart from all other ideas, he would fall asleep. For our own part, we think such a plan more likely to keep a nervous person awake. The best hypnotic we know, especially for those engaged in brain-work, is a cold sponge all over just before going to bed. The sudden chill, followed by the warmth of the bed-clothes, seems to relieve the congested condition of the vessels of the brain and thus induce sleep.

